# Comprehensive 90‐Day Morbidity Assessment Following Robot‐Assisted Radical Cystectomy With Intracorporeal Diversion

**DOI:** 10.1155/aiu/5585494

**Published:** 2025-12-29

**Authors:** Anas Elyan, Laura Wimmer, Jan Ebbing, Helge H. Seifert, Abolfazl Hosseini, Ashkan Mortezavi

**Affiliations:** ^1^ Department of Urology, University Hospital of Basel, Basel, Switzerland, unispital-basel.ch; ^2^ Department of Urology, Danderyd University Hospital, Stockholm, Sweden; ^3^ Department of Urology, University Hospital of Zurich, Zurich, Switzerland, usz.ch

**Keywords:** Clavien–Dindo classification, enhanced recovery after surgery (ERAS), intracorporeal urinary diversion (ICUD), perioperative complications, robot-assisted radical cystectomy (RARC), systemic vs. surgical morbidity, ureteral stricture

## Abstract

**Objective:**

To evaluate perioperative morbidity following robot‐assisted radical cystectomy with intracorporeal urinary diversion (iRARC) during the transition from open surgery at a single academic center, and to provide detailed complication profiles to guide surgical teams and patient counseling.

**Patients and Methods:**

We retrospectively analyzed 111 consecutive patients undergoing iRARC between 2017 and 2023. Perioperative outcomes and complications were assessed at 30 and 90 days, using the Clavien–Dindo (CD) classification. Complications were further categorized by etiology and severity. Detailed incidence rates and management strategies were recorded per patient.

**Results:**

A total of 163 complications occurred within 90 days, with 129 (79%) recorded within 30 days. Early complications were predominantly low‐grade (*n* = 96, 74%), while 33 (26%) were high‐grade. Systemic complications 108 (84%) outnumbered primary surgical events 21 (16%). Among early high‐grade complications, cardiovascular and respiratory events were the most common (*n* = 9, 27%), followed by infectious (*n* = 7, 21%) and genitourinary complications (*n* = 7, 21%). Incidence rates for events were calculated on a per‐patient basis: acute coronary syndrome 1.2%, pneumonia 2.7%, arrhythmias 2.7%, thrombosis 1.8%, pulmonary embolism 0.9%, insufficiency of the ileal anastomosis 0.9%, urinary tract infections 32.4%, and infected lymphoceles 8.1%. Genitourinary complications, including ureteral strictures (6.3%) and urinary leakage (3.6%), required frequent endourological interventions. Despite minimally invasive surgery and strict ERAS protocol implementation, paralytic ileus occurred in 19.8% of patients but was managed conservatively in all cases. Thirty‐four additional complications occurred between days 30 and 90, including 16 high‐grade events. The 90‐day mortality rate was 1.8%. In the multivariable analysis, no independent predictors of high‐grade complications were identified. Variables including BMI, clinical stage ≥ cT2, age, sex, and neoadjuvant chemotherapy showed no significant association (all *p* ≥ 0.20).

**Conclusion:**

iRARC is feasible and associated with an acceptable safety profile during the learning curve. Most complications were systemic and not directly related to the surgical technique, underscoring the need for multidisciplinary perioperative management. Detailed incidence data of specific complications provide valuable insights for realistic patient counseling and heightened awareness among care teams.

## 1. Introduction

Bladder cancer (BCa) poses a significant health risk globally, functional and oncological outcomes, and imposes a substantial socioeconomic burden [1]. Its incidence is steadily rising, and it now ranks as the 10th most common cancer worldwide [2]. Radical cystectomy (RC) accompanied by pelvic lymphadenectomy (PLND) stands as the established standard of care for managing muscle‐invasive and very high‐risk BCa [3]. However, due to the extensive nature of the procedure and the high prevalence of comorbidities in this population, RC is associated with high rates of perioperative morbidity and mortality [4]. Minimally invasive approaches, aiming to reduce surgical morbidity and shorten hospital stays, have gained widespread adoption. Robot‐assisted RC (RARC) has emerged as a viable option since 2003, with a notable increase in its utilization over the years [5]. Initially, a hybrid approach with extracorporeal urinary diversion (ECUD) was used. Most RARCs are now performed using intracorporeal urinary diversion (ICUD) in high‐volume centers [6].

Recent studies have demonstrated that RARC offers oncological and perioperative outcomes comparable to those of open cystectomy [7]. Additionally, RARC provides potential advantages, including reduced blood loss, lower transfusion rates, diminished postoperative pain, faster bowel recovery, and shorter hospital stays [8, 9]. However, performing urinary diversion with robot‐assisted laparoscopy remains technically challenging and requires a learning curve, similar to other complex surgical procedures [10]. While the number of cases needed to achieve proficiency in key outcome parameters has been explored, there is limited knowledge of the specific complications associated with ICUD during the adoption phase. A detailed understanding of the nature and frequency of potential adverse events is essential not only for optimizing their management by the medical team but also for ensuring patients receive accurate and thorough preoperative counseling.

To address this gap, we conducted an in‐depth analysis of complications occurring within the first 90 days after RARC with ICUD (iRARC) in a consecutive case series during our transition from an open to a fully robot‐assisted approach.

## 2. Patients and Methods

### 2.1. Patients

Prospective data were collected systematically on consecutive patients undergoing RC between April 2017 and April 2023 at a single academic institution. These data were maintained in an institutionally approved database. The cohort included patients undergoing RC for muscle‐invasive BCa, (very) high‐risk non‐muscle‐invasive BCa, and nonmalignant bladder diseases. Prior to this period, all RCs were performed via an open approach (ORC). Initially, iRARC was offered to selected patients based on factors such as body mass index (BMI), tumor stage, prior abdominal surgery, and occasionally also on operating‐room capacity. As experience grew, all consecutive cases were scheduled for iRARC. Details of this transition are provided in Supporting Table 1.

All iRARC procedures were conducted by three designated surgeons experienced in ORC and robot‐assisted radical prostatectomy. Outcome was assessed in all patients scheduled for iRARC (intention‐to‐treat). Patients undergoing palliative cystectomy due to locally advanced disease or metastatic disease were excluded.

### 2.2. Surgical Approach

RARC with ICUD was performed as previously described [11]. Perioperative antibiotic prophylaxis consisted of single‐shot second‐generation cephalosporin and metronidazole. A standard six‐port transperitoneal approach was used, with the patient positioned at a 26° Trendelenburg angle. We used the da Vinci® Si and Xi robotic system (Intuitive Surgical, Sunnyvale, CA, USA). Pelvic lymph node dissection (PLND) in malignant disease encompassed external, internal, and common iliac as well as presacral and obturator lymph nodes. However, in elderly patients and non‐muscle‐invasive disease removal of presacral nodes was omitted. Cystectomy was performed using an endoscopic vessel sealer device operated by the bedside assistant (LigaSure™). Bilateral nerve‐sparing was performed in all men receiving an orthotopic bladder substitution (OBS) and in selected men with sufficient erectile function receiving an ileal conduit (IC). The left ureter was passed posteriorly to the sigmoid mesocolon to the right. Construction of the IC involved mobilizing a 15‐cm segment of terminal ileum located 20 cm from the ileocecal valve using a laparoscopic 60 mm (vascular/medium) stapler (Endo‐GIA, Covidien Corp., Dublin, Ireland). For continent diversion, a 55‐cm segment of ileum was utilized and an OBS constructed according to the Studer technique. Ureteroileal anastomosis for both the IC and OBS was performed using the Wallace technique with a PDS 5.0 suture. Single‐J ureteral stents were placed in all patients, and a drain was used only in those with OBS. Perioperative management was based on an enhanced recovery program (ERP). Single‐J stent was removed on the 10th postoperative day (POD). Thromboprophylaxis was provided with unfractionated heparin for 4 weeks postoperatively. A full description of the ERP and timings is provided in Supporting Table 2.

### 2.3. Patient Demographics and Outcome

Patient demographics included age, sex, BMI, American Society of Anesthesiologists (ASA) score, clinical T‐stage, and receipt of neoadjuvant chemotherapy (NAC). Our primary aim was to perform a detailed analysis of postoperative complications including the 30‐day reoperation and rehospitalization rate. Secondarily, we aimed to assess perioperative parameters such as transfusion rate, operation time, and length of stay (LOS). Additional perioperative parameters collected included the performance of PLND, the number of removed lymph nodes, nodal status, pathological T‐stage, and diversion type classified as IC or OBS. Cases of iRARC converted to open surgery were included in the iRARC cohort for analysis.

### 2.4. Complications

This study adheres to the European Association of Urology (EAU) guidelines recommendations on reporting and grading of complications [12]. For further details and a checklist, see Supporting Table 3. All intraoperative and postoperative complications were classified using the modified Clavien–Dindo (CD) system [12]. These complications were further categorized by etiology into the following groups: gastrointestinal, cardiovascular and respiratory, infectious, abdominal wall and stoma, genitourinary, neurologic, metabolic, death, and other causes. Paralytic ileus, categorized under the gastrointestinal group, was defined as persistent postoperative vomiting requiring nasogastric tube insertion or the inability to tolerate enteral intake for 48 h or longer. Metabolic acidosis was defined as decreased blood pH requiring bicarbonate therapy. Events involving both ureteric stricture and pyelonephritis were classified as complications attributable to the ureteric stricture. Urosepsis was defined as a life‐threatening organ dysfunction caused by a urological infection, characterized by an increase in the SOFA score of ≥ 2 points from baseline (Sepsis‐3). We distinguished between primary surgical complications directly attributable to the surgical technique and systemic postoperative complications arising from the surgical process. Incidence rates based on per patient analysis were calculated for 90 days.

### 2.5. Trifecta Status

In line with contemporary approaches to reporting surgical quality, we incorporated a modified trifecta assessment based on the framework as described in previous work [13]. Our proposed definition included negative surgical margins, removal of at least 16 lymph nodes, and absence of high‐grade complications within 90 days postoperatively.

### 2.6. Statistics

Continuous variables are reported as medians with interquartile ranges (IQR), and categorical variables as absolute numbers and percentages. Group comparisons were performed using the *χ* ^2^ test or Fisher’s exact test for categorical variables and the Mann–Whitney *U* test for continuous variables. Variables with a *p* value < 0.10 in univariate analysis were entered into a multivariate logistic regression model to explore potential independent predictors of high‐grade complications.

The analysis was exploratory, and no formal power calculation was performed. Statistical significance was defined as a two‐sided *p* value < 0.05. All analyses were conducted using R Statistical software (v4.1.2; R Core Team 2021).

## 3. Results

Between February 2017 and December 2023, 158 RCs were performed. After excluding 47 open RCs (ORCs), three palliative iRARCs, and one iRARC without urinary diversion, 111 iRARC cases (70%) were analyzed (see flowchart, Supporting Figure 1). The median patient age was 72 years (IQR: 64–78), with 92 (83%) being men. BCa was the primary indication in 103 cases (93%), while five (5%) had neurogenic bladder and three (3%) interstitial cystitis. The median BMI was 25 kg/m^2^ (IQR: 23–29). Most patients (91%, *n* = 101) had an ASA score ≥ III, with 9 (8%) classified as ASA I‐II. Baseline characteristics and perioperative outcomes are summarized in Tables 1 and 2, respectively.

**Table 1:** Baseline characteristics of the overall patient cohort (*n* = 111).

Variables	Overall cohort (*n* = 111, 100%)
Baseline	
Age, median (IQR), year	72 (64–78)
Gender, no. (%)	
Male	92 (83%)
Female	19 (17%)
BMI, median (IQR), kg/m²	25 (23–29)
Bladder cancer, no. (%)	103 (93%)
Neoadjuvant chemotherapy, no. (%)	38 (34%)
ASA‐score, no. (%)	
I	1 (1%)
II	8 (7%)
III	101 (91%)
IV	1 (1%)
Clinical T stage, no. (%, denominator 103)	
CIS/Ta/T1	39 (38%)
T2	59 (57%)
T3	4 (4%)
T4	1 (1%)

Abbreviations: ASA = American Society of Anesthesiologists physical status classification system, BMI = body mass index, CIS = Carcinoma in situ, IQR = interquartile range.

**Table 2:** Perioperative outcome parameters of the overall patient cohort (*n* = 111).

Variables	Overall cohort (*n* = 111, 100%)
Peri‐ and postoperative outcomes	
Deviation type, *n* (%)	
Ileal conduit	91 (82%)
Orthotopic bladder substitution	20 (18%)
Blood transfusion, *n* (%)	23 (21%)
Operation time, median (IQR), minutes	408 (345–421)
Removed lymph nodes^∗^, median (IQR)	18 (12–24)
Pathological T‐stage, *n* (%)	
CIS/Ta/T1/T0	49 (48%)
T2	27 (27%)
≥ T3	20 (20%)
T4	6 (6%)
*N*+^∗^, *n* (%)	11 (11%)
Length of stay, median (IQR), days	15 (13–21)
Reoperation (0–30 days), *n* (%)	14 (8%)
Rehospitalization (0–30 days), *n* (%)	24 (22%)
30 d complications, *n* (%)	89 (80%)
Clavien grade 1‐2	63 (57%)
Clavien grade 3–5	26 (23%)
30–90 d complications, *n* (%)	33 (30%)
Clavien grade 1‐2	19 (17%)
Clavien grade 3–5	14 (13%)

*Note:* d = days, *N*+ = presence of regional lymph node metastasis.

Abbreviations: CIS = Carcinoma in situ; IQR = interquartile range.

^∗^In patients undergoing pelvic lymph node dissection.

### 3.1. Primary Outcome

Within 90 days postoperatively, 163 complications were recorded (Table 3), with 129 (79%) occurring within 30 days. Of these early events, 96 (74%) were low‐grade (CD Grades I–II) and 33 (26%) high‐grade (CD grades IIIa–IV). Per patient, 75 (68%) had low‐grade complications, and 26 (23%) experienced high‐grade early complications. Primary surgical complications within 30 days accounted for 21 (16%) of 129 events, including urinary leakage (*n* = 4), ureteral stricture (*n* = 2), wound dehiscence/abscess (*n* = 3), fascial dehiscence (*n* = 1), infected lymphocele (*n* = 5), and bleeding requiring transfusion (*n* = 6). Systemic complications constituted 108 (84%) of cases, predominantly infections.

**Table 3:** Subclassification of reported complications in the overall cohort.

	*N*	(%)
All reported complications	163	100.0
Gastrointestinal	25	14.9
Paralytic ileus	22	13.1
Ileal anastomotic leak	1	0.6
Melena	1	0.6
Upper gastrointestinal bleeding	1	0.6
Cardiovascular and respiratory	29	17.3
Anemia	8	4.8
Pneumonia	4	2.4
Vasopressor‐dependent hypotension	4	2.4
Atrial fibrillation	3	1.8
Acute coronary syndrome^1^	2	1.2
Deep vein thrombosis	2	1.2
Acute respiratory distress syndrome	1	0.6
COPD exacerbation	1	0.6
Pulmonary thromboembolism	1	0.6
Prolonged postoperative ventilation due to upper airway edema	1	0.6
Pulmonary edema	1	0.6
Percutaneous coronary intervention	1	0.6
Infectious complications	56	33.3
Urinary tract infection	37	22.0
Infected lymphocele	9	5.4
Nonobstructive pyelonephritis	3	1.8
Wound infection	3	1.8
Obstructive pyelonephritis	1	0.6
Urosepsis	1	0.6
Intra‐abdominal abscess	1	0.6
Multiorgan failure	1	0.6
Abdominal wall and stoma	2	1.2
Fascial dehiscence	1	0.6
Inguinal hernia	1	0.6
Genitourinary	24	14.3
Ureteral stricture (unilateral)	6	3.6
Ureteral stricture (bilateral)	1	0.6
Urinary leakage (ureter and urethra)	6	3.6
Recatheterization of neobladder	2	1.2
Ureteral catheter malposition	2	1.2
Ureteral catheter replacement	3	1.8
Ureter perforation	1	0.6
Requirement for pyelography	1	0.6
Requirement for ureterorenoscopy	1	0.6
Requirement for loopography	1	0.6
Neurologic disorders	6	3.6
Hyperactive delirium	2	1.2
Ischemic stroke	2	1.2
Decreased level of consciousness	1	0.6
Radicular leg pain	1	0.6
Metabolic acidosis	5	3.0
Death	2	1.2
Other	2	1.2
Anaphylactic shock	1	0.6
Acute bleeding	1	0.6
Unplanned pharmacological interventions	12	7.1
Potassium supplementation	8	4.8
Antibiotic eye drops	1	0.6
Antihistamines	1	0.6
Sodium substitution	1	0.6
Opioid substitution therapy	1	0.6

*Note:* The table presents the distribution of all reported complications categorized by etiology in patients undergoing robotic cystectomy with intracorporeal urinary diversion.

Abbreviation: COPD = chronic obstructive pulmonary disease.

^1^ST‐elevation myocardial infarction and non‐ST‐elevation myocardial infarction.

### 3.2. 30–90‐Day Complications

Between 30 and 90 days after surgery, 34 complications were registered. Of these, 18 (53%) were classified as low‐grade and 16 (47%) as high‐grade. Primary surgical complications were observed in 13 events (38%) and systemic in 21 (62%) events. Infections (*n* = 15, 44%) were with ureteral stricture (*n* = 5, 15%) and lymphoceles (*n* = 4, 12%), the most common adverse event of the recorded complications beyond 30 days.

### 3.3. High‐Grade Complications

Among early high‐grade complications (*n* = 33), cardiovascular and respiratory events were the most common (*n* = 9, 27%), followed by infectious (*n* = 7, 21%) and genitourinary complications (*n* = 7, 21%). Among late high‐grade complications (*n* = 16), genitourinary (*n* = 8, 53%) and infectious (*n* = 5, 33%) events accounted for the majority of cases. A detailed analysis of early and late high‐grade complications, along with their respective management strategies, is provided in Tables 4 and 5.

**Table 4:** Subclassification of high‐grade (Clavien–Dindo ≥ 3a) complications occurring within 30 days postoperatively.

	*n*	%	Clavien–Dindo grade	Intervention/justification
All reported complications	33			
Gastrointestinal	3	9.1		
Ileal anastomotic leak	1	3.0	3b	Under general anesthesia, a laparotomy was performed due to an ileal anastomotic leak and patient underwent surgical treatment.
Melena	1	3.0	3a	Gastroscopy was performed. Multiple ulcers of the pylorus and duodenal bulb were found as the cause and were treated with proton pump inhibitors.
Upper gastrointestinal bleeding	1	3.0	3a	Gastroscopy was performed due to an unexplained decrease in hemoglobin. Duodenal ulcers were identified as the source of bleeding, and treatment with proton pump inhibitors was initiated.
Cardiovascular and respiratory	9	27.3		
Pneumonia	1	3.0	4a	Severe pneumonia, most likely aspiration‐related, requiring bronchoscopy and intensive care unit (ICU) management.
Pneumonia	1	3.0	3a	Bronchoscopy was indicated.
Hypotension requiring vasoactives	3	9.1	4a	Vasoactive treatment in the ICU was required in three cases due to the following conditions: NSTEMI following surgery, undifferentiated shock, and ARDS.
Acute coronary syndrome	2	6.1	4a	Two patients experienced NSTEMIs requiring intensive care unit admission.
ARDS	1	3.0	4a	ICU admission was required.
Exacerbated COPD	1	3.0	4a	Postoperatively exacerbated COPD requiring ICU care.
Infectious complications	7	21.2		
Infected lymphocele	4	12.1	3a	CT‐guided drainage under local anesthesia was required.
Wound infection	1	3.0	4a	Due to a wound infection, surgical exploration under general anesthesia was performed, revealing a small intestinal fistula. ICU admission was required.
Urosepsis	1	3.0	4a	ICU admission was required.
Intra‐abdominal abscess	1	3.0	3a	CT‐guided drainage under local anesthesia was required.
Abdominal wall and stoma	2	6.1		
Fascial dehiscence	1	3.0	3b	Laparotomy with hernia repair using sublay mesh placement.
Inguinal hernia	1	3.0	3b	Unilateral inguinal hernia repair performed for incarcerated hernia.
Genitourinary complications	7	21.2		
Ureteral stricture (unilateral)	1	3.0	3a	Nephrostomy placement under local anesthesia.
Ureteral stricture (unilateral)	1	3.0	3b	Nephrostomy placement under general anesthesia.
Urinary leakage	1	3.0	3a	Due to insufficiency of the left ureteral anastomosis, a nephrostomy was placed under local anesthesia.
Urinary leakage	1	3.0	4a	Laparotomy with small bowel repair was performed for an anastomotic leak, along with internal hernia reduction and closure of the mesenteric defect. ICU admission was required.
Urinary leakage	1	3.0	3b	A urinoma caused by leakage at the ureteroenteric anastomosis into the ileal conduit was treated with bilateral nephrostomy placement under local anesthesia. In addition, pigtail drainage was inserted by interventional radiologists under local anesthesia.
Urinary leakage	1	3.0	4a	Wallace plate revision was performed due to a minor leak, including partial oversewing of the ureteral conduit anastomosis. Postoperatively, ICU monitoring was required.
Ureter perforation	1	3.0	3b	Intraoperative perforation of the right proximal ureter occurred during Mono‐J catheter insertion due to ureteral kinking, necessitating reoperation under general anesthesia.
Neurologic disorders	3	9.1		
Hyperactive delirium	1	3.0	4a	ICU admission was required.
Ischemic stroke	1	3.0	4a	An unplanned ICU stay was necessary due to a subacute infarction in the left cerebral hemisphere, most likely cardioembolic in origin, caused by an occlusion in a branch of the posterior cerebral artery.
Reduced vigilance	1	3.0	4a	ICU admission was required. The underlying etiology remained unclear, with differential diagnoses including urosepsis and postoperative benzodiazepine effects. Patient recovered quickly.
Death	1	3.0	5	The patient developed asystole, and resuscitation efforts were unsuccessful. No autopsy was performed.
Other	1	3.0		
Emergency laparotomy	1	3.0	4a	An emergency laparotomy was performed on the evening of the initial surgery due to arterial bleeding from the mesenteric branch of the conduit. The procedure, carried out under general anesthesia, included hemostatic control, evacuation of blood clots, abdominal lavage, and placement of a drain.

*Note:* All high‐grade complications within 30 days are listed according to etiology. Additionally, the corresponding Clavien–Dindo grading is provided along with the reason for the grading. If complications and their causes were repetitive, they are summarized in one line; otherwise, multiple entries were made.

Abbreviations: ARDS = acute respiratory distress syndrome, COPD = chronic obstructive pulmonary disease, CT = computed tomography, ICU = intensive care unit, NSTEMI = non‐ST‐elevation myocardial infarction.

**Table 5:** Subclassification of high‐grade (Clavien–Dindo ≥ 3a) complications occurring within 30–90 days postoperatively.

	*n*	%	Clavien–Dindo grade	Intervention/justification
All reported complications	16			
Cardiovascular and respiratory	1	6.3		
Pneumonia	1	6.3	4a	Rehospitalization occurred due to bilateral pneumonia, managed with intravenous antibiotics. ICU admission was required for monitoring, bronchoscopy, and thoracoscopic wedge resection of the lower lung lobe.
Infectious complications	5	31.3		
UTI	1	6.3	4a	Urosepsis secondary to infection, requiring intensive care unit (ICU) admission.
Infected lymphocele	3	18.8	3a	CT‐guided drainage performed under local anesthesia.
Multiorgan failure	1	6.3	4b	Rehospitalization due to multiorgan failure, including septic shock of unclear etiology, septic encephalopathy, respiratory failure with hypercapnia, and acute‐on‐chronic kidney injury.
Genitourinary complications	8	50.0		
Ureteral stricture (unilateral)	1	6.3	3a	Unplanned readmission for left percutaneous nephrostomy placement under local anesthesia due to newly diagnosed grade 3 hydronephrosis of the left kidney.
Ureteral stricture (unilateral)	1	6.3	3a	Nephrostomy placed under local anesthesia due to dislocated Mono‐J stent and pre‐existing ureteral stricture.
Ureteral stricture (unilateral)	1	6.3	3a	Due to impaired urinary outflow, a double‐J stent was placed from the ileal conduit into the left kidney.
Ureteral stricture (unilateral)	1	6.3	3b	Left‐sided urinary transport disorder required left nephrostomy and double‐J stent insertion under general anesthesia, following failed Mono‐J catheter placement within the first 30 postoperative days.
Ureteral stricture (bilateral)	1	6.3	3b	Nephrostomy was performed under general anesthesia due to right‐sided urinary outflow obstruction. Additionally, the double‐J stent on the contralateral side was exchanged under local anesthesia.
Urinary leakage	1	6.3	3b	Nephrostomy was placed under general anesthesia due to a febrile urinary tract infection, with CT evidence of contrast extravasation near the left ureter.
Ureterorenoscopy	1	6.3	3b	Further diagnostics were required due to ureteral stricture.
Insufficiency of anastomosis	1	6.3	3b	Retrograde bilateral placement of double‐J stents was performed during looposcopy due to complete dislocation of both stents, with leakage at the Wallace plate and impaired drainage on the left.
Death	1	6.3	5	Long‐segment small bowel perforation following surgery.
Other	1	6.3		
Anaphylactic shock	1	6.3	4a	The patient developed anaphylactic shock in response to the administered antibiotic therapy.

*Note:* All high‐grade complications within 30–90 days are listed according to etiology. Additionally, the corresponding Clavien–Dindo grading is provided along with the reason for the grading. If complications and their causes were repetitive, they are summarized in one column; otherwise, multiple entries were made.

Abbreviations: ARDS = acute respiratory distress syndrome, COPD = chronic obstructive pulmonary disease, CT = computed tomography, ICU = intensive care unit, NSTEMI = non‐ST‐elevation myocardial infarction.

### 3.4. Predictors of High‐Grade Complications

No predictor reached statistical significance; effect estimates were near null for age, BMI, sex, operation time, stage, and NAC (Supporting Table 4). ASA was not modeled due to near‐zero variance.

### 3.5. Comprehensive Per Patient Analysis of Complications (0–90 Days)

#### 3.5.1. Cardiovascular and Respiratory Complications

An acute coronary syndrome in our patient population was observed in two cases (incidence rate 1.2%), both managed successfully in collaboration with cardiologists.

#### 3.5.2. Case Presentation

Shortly after iRARC (operative time: 345 min), a male patient presented with ST elevations on ECG and elevated troponin levels, consistent with NSTEMI. Transthoracic echocardiography (TTE) revealed wall motion abnormalities, and coronary angiography identified severe occlusion of the distal left main coronary artery, necessitating coronary artery bypass grafting. The patient was admitted to the intensive care unit (ICU).

The patient’s medical history included AV‐nodal re‐entry tachycardia, successfully treated with radiofrequency ablation 5 years prior. He had no cardiovascular risk factors, was a nonsmoker, and had no preoperative anemia or chronic kidney disease. He had received four cycles of NAC with Cisplatin/Gemcitabine and Durvalumab.

Due to concurrent infectious and urological complications, including urinary leakage, cardiac bypass surgery was delayed and successfully performed 4 weeks postoperatively. The subsequent clinical course was uneventful.

Three patients developed pneumonia (incidence rate 2.7%).

### 3.6. Case Presentation

A male patient underwent iRARC (operative time: 430 min) for BCa, initially without complications. However, paralytic ileus‐induced vomiting on POD 6 preceded progressive atypical pneumonia, culminating in ICU transfer on POD 10 for suspected ARDS. CT revealed pulmonary infiltrates, consolidation, and bilateral pleural effusions. Prednisone therapy was initiated, and *Klebsiella pneumoniae* was identified in bronchoalveolar lavage, prompting targeted Tazobactam therapy. Pleural punctures confirmed Gram‐negative rods. The patient was weaned from high‐flow oxygen to BiPAP and later stabilized on nasal cannula oxygen. He made a full recovery without further complications.

Three patients developed postoperative cardiac arrhythmias (incidence rate 2.7%). All three cases presented as new‐onset atrial fibrillation on POD 4, 5, and 11, managed with oral Rivaroxaban. While two cases were oligosymptomatic, one fibrillation was associated with ARDS.

Postoperative thrombosis occurred in two patients (incidence rate 1.8%). Both cases presented as deep vein thrombosis (DVT). The first case was diagnosed on POD 29, two days after cessation of thrombosis prophylaxis, presenting as lower limb redness. The second case, diagnosed on POD 5, involved thrombosis of the subclavian vein associated with a port catheter. Both were started on apixaban.

One patient developed a pulmonary embolism, detected via CT after desaturation at POD 5 (incidence rate 0.9%). Heparin therapy was initiated, with no further intervention needed due to hemodynamic stability. The embolus was presumed parainfectious, associated with active bilateral pneumonia.

### 3.7. Gastrointestinal Complications

Among the gastrointestinal complications, paralytic ileus was the most common complication, typically occurring on the third POD. These events were classified as CD Grade 2 (low‐grade) with an incidence rate of 19.8% (*n* = 22) in our study population. Patients showing symptoms such as nausea, constipation, and a distended abdomen were placed on nil per os status. If vomiting occurred, a nasogastric tube was inserted to relieve pressure on the gut and to prevent aspiration pneumonia.

The anastomotic leak of the ileal segment occurred in a 69‐year‐old male who underwent iRARC with IC (operation time: 421 min) for BCa, resulting in an incidence rate of 0.9%. A CT scan performed on POD 8 due to fever revealed possible infected hematoseroma. The patient was treated with further antibiotics. However, a follow‐up CT scan at day 12 revealed anastomotic insufficiency. The patient underwent emergency laparotomy with oversewing of the ileal anastomosis.

Two patients experienced gastrointestinal bleeding (incidence rate 1.8%), presented with melena and hematemesis on POD 6 and 8. Both patients underwent gastroscopy and colonoscopy, which revealed upper duodenal lesions without active bleeding. Both were managed conservatively with proton pump inhibitors.

### 3.8. Infectious Complications

Infectious complications occurred in 49 patients (incidence rate: 44.1%) with urinary tract infections (UTIs) being most common (36 patients, incidence rate: 32.4%). All but one case were classified as CD Grade II (low‐grade).

Infected lymphoceles were identified in nine patients (incidence rate of 8.1%), leading to seven high‐grade complications requiring intervention (incidence rate of 6.3%). The median time to symptom onset—typically characterized by persistent fever despite antibiotic treatment, abdominal tenderness in the lower quadrants, and elevated infection markers—was 11.5 days postoperatively. Diagnosis was confirmed via CT, and CT‐guided drainage was performed subsequently in 7 patients. Two patients were managed conservatively with IV antibiotics.

Pyelonephritis occurred in four cases (incidence rate 3.6%), three nonobstructive and one obstructive due to a single‐J catheter occlusion, which was replaced under local anesthesia.

### 3.9. Genitourinary Complications

The most common genitourinary complication was ureteral stricture, occurring in seven patients (incidence rate 6.3%).

#### 3.9.1. Case Presentation

**Figure 1:**
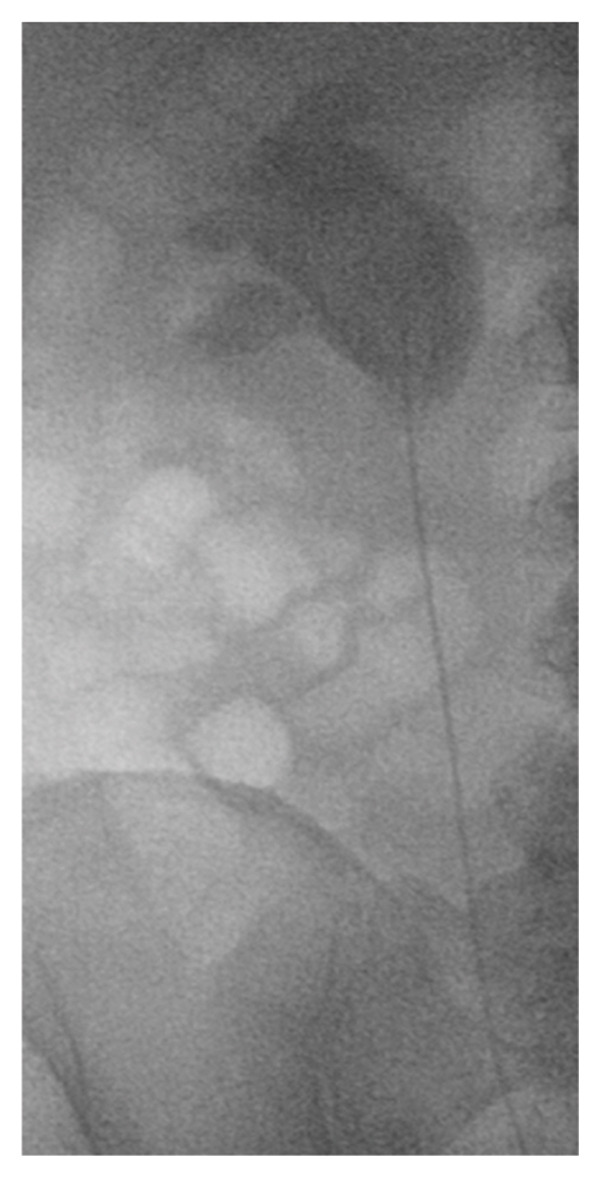
Retrograde pyelography demonstrated contrast outlining a blood clot within the renal pelvis. Regular flushing of the renal pelvis was performed.

A 59‐year‐old male with BCa underwent iRARC with creation of an OBS (operation time 457 min). Discharged on POD 8 with a single‐J stent, he was readmitted with fever and stent dislocation. Antibiotic treatment was initiated. Due to rising creatinine and progressive ectasia of the right kidney, a nephrostomy was placed on POD 17. Antegrade pyelography revealed normal urinary outflow and the nephrostomy was removed on the POD 22. Following nephrostomy removal, the patient experienced severe arterial bleeding from the puncture site and a bladder tamponade. CT angiography revealed an arteriovenous fistula, successfully treated with emergency coiling. A transurethral clot removal was performed under general anesthesia. Due to flank pain a CT was performed 2 days later revealing blood clots in the renal pelvis obstructing urinary outflow. Regular flushing of the renal pelvis with a newly placed single‐J stent was performed (Figure 1). Despite ongoing single‐J stent drainage, persistent clots seen on CT required percutaneous nephrolithotomy for clot removal 6 months later. Post‐procedure, the nephrostomy was removed, and the patient remained free of further obstruction.

Urinary leakage occurred in four cases (incidence rate 3.6%), all associated with conduits. Typical symptoms included abdominal pain and elevated creatinine levels. In one case, an occluded ureteral catheter was considered a potential etiology. One leakage was diagnosed beyond 30 days and was associated with obstruction. All but one case was managed conservatively with ureteral catheters and nephrostomies. One case required laparotomy due to worsening abdominal pain and suspicion of an internal hernia on CT imaging. However, laparotomy did not confirm an internal hernia but instead revealed a small urinary leak at the Wallace plate, which was successfully sutured.

### 3.10. Other Complications

A CD 4a complication occurred in a patient who initially underwent IC formation for T2 BCa. Postoperatively, the patient required an emergency laparotomy due to arterial bleeding from the mesentery. Hemostasis was achieved by ligating the bleeding branch supplying the conduit and evacuating the blood clots. The patient required transfusion of two red blood cell units.

**Figure 2:**
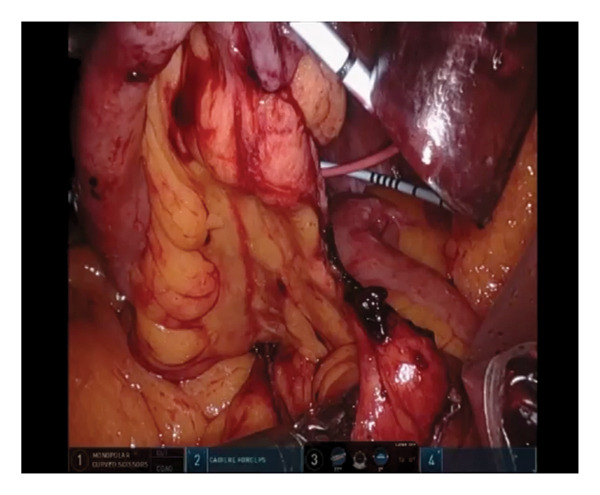
Intraoperative imaging from the iRARC indicated mesenteric bleeding.

Subsequent urinary leakage at the ureteral anastomosis site necessitated bilateral percutaneous nephrostomy placement. A retrospective review of the surgical video revealed that the mesenteric bleeding could potentially have been identified earlier in the procedure (Figure 2).

### 3.11. Secondary Outcome

ICUD with IC was the preferred modality and was performed in 91 patients (82%), and 20 patients (18%) received an OBS. The median operating time was 408 min (IQR: 345–421), with a median LOS of 15 days (IQR: 13–21). Rehospitalization occurred in 24 patients (22.3%), and the red blood cell transfusion was administered in 23 patients (20.7%). Detailed perioperative outcomes are outlined in Table 2. The overall 90‐day mortality rate was 1.8% (*n* = 2), with one patient developing sudden cardiac arrest with unknown cause and the second patient developing wound infection with small intestine fistula and death on day 30 postoperatively after switching to best supportive care. This case was converted to open surgery because port placement was not possible due to severe intestinal adhesions. Applying the trifecta definition (negative surgical margins, retrieval of ≥ 16 lymph nodes, and absence of CD ≥ IIIa complications within 90 days), 38 of 92 evaluable patients (41.3%) fulfilled all three criteria [14].

## 4. Discussion

In this single‐center experience, we provide a comprehensive assessment of 90‐day morbidity after iRARC. The findings underscore both the safety and the challenges of implementing this complex technique in routine practice, including during the transition from open to robotic surgery. A core observation is that most complications within the first 30 days were systemic rather than technical. Only 16% were attributable directly to surgery, whereas infectious, cardiovascular, and respiratory events predominated—together representing 27% of early high‐grade complications. This emphasizes that morbidity after iRARC is driven largely by patient‐related and systemic factors and highlights the need for rigorous perioperative management, particularly in patients with significant comorbidities.

Importantly, the present cohort reflects the early institutional transition from open to fully intracorporeal robotic cystectomy. Although individual surgeon experience contributes to outcomes, interpreting the data purely as a surgeon‐level learning curve would be misleading. During the study period, operative responsibility was shared across fellowship‐trained surgeons and externally proctored cases, resulting in a balanced annual case distribution that prevents meaningful attribution of outcomes to single operators. Moreover, several of the most frequent complications—such as cardiopulmonary events and infections—are primarily center‐dependent and relate to perioperative pathways, infrastructure, and multidisciplinary workflows rather than to the technical proficiency of a single surgeon. For these reasons, the findings should be understood as representative of the institutional learning curve and the maturation of center‐wide processes required to safely implement intracorporeal RARC, rather than as outcomes driven by individual surgeon growth.

The present analysis complements the evidence generated by the two available randomized trials comparing RARC with open surgery. The iROC trial demonstrated modest early recovery benefits for RARC with intracorporeal diversion, including more days alive and out of hospital and fewer wound and thromboembolic complications, while showing no significant differences in overall complication rates or oncologic outcomes [8]. The randomized study by Mastroianni et al. further supports the equivalence of iRARC to open cystectomy in terms of surgical quality [13]. That trial found no differences in USC pentafecta or trifecta achievement, confirming comparable oncologic and perioperative quality benchmarks between approaches when performed by experienced teams. A direct comparison of our findings with the iRARC arms of these trials is only partly feasible for several reasons. First, despite their prospective design, selection bias cannot be excluded. Patients enrolled in the iROC trial and in the study by Mastroianni et al. were notably younger (mean age 69 and 62 years, respectively) than those in our cohort (72 years). Additionally, in the latter trial, 77% of patients had an ASA score of 1–2, whereas 91% of our patients had an ASA score of 3–4. Our cohort also included individuals undergoing cystectomy for benign disease. Moreover, the present data reflect the implementation phase of a newly introduced technique at a single institution, which further limits comparability with established trial cohorts. Taken together, our series represents a real‐world population of older, more comorbid patients, which likely contributes to the higher complication rates observed (23% at 30 days vs. 17% at 30 days and 16% at 90 days in the cited trials). In contrast to the aggregated reporting of the RCTs, our data during program implementation provides a detailed complication profile, showing that postoperative morbidity after iRARC is driven largely by systemic events—particularly infectious, cardiopulmonary, and gastrointestinal complications—while true surgical failures such as ureteroenteric strictures or urinary leakage were less frequent and comparable to contemporary intracorporeal diversion series [15, 16]. This combined perspective underscores that optimizing outcomes after cystectomy requires not only technical proficiency in intracorporeal diversion but also robust multidisciplinary perioperative care focused on infection prevention, cardiopulmonary management, and early gastrointestinal recovery.

A notable strength of our study is the provision of detailed incidence rates for individual complications—many of which have not been systematically reported before in the context of iRARC with intracorporeal diversion. These include, for example, paralytic ileus (19.8%), Ileal anastomotic leak (0.9%), acute coronary syndrome (1.2%), cardiac arrhythmias (2.7%), pneumonia (2.7%), pulmonary embolism (0.9%), urinary leakage (3.6%), and infected lymphoceles (8.1%). The availability of these data enables a more realistic and evidence‐based preoperative counseling of patients. This fosters shared decision‐making and may help manage patient expectations more effectively. Moreover, awareness of these incidence rates equips the multidisciplinary surgical and perioperative care team to remain particularly vigilant for early signs of these complications, ensuring timely intervention. Acute coronary syndromes, arrhythmias, and pneumonia accounted for a significant proportion of early high‐grade complications, some of which may lead to irreversible damage. These events warrant the immediate involvement of nonsurgical specialties to ensure timely diagnosis and multidisciplinary management.

Infectious complications were the most frequent adverse events (44.1%), predominantly low‐grade UTIs. The intracorporeal technique—particularly limited ability to cleanse the conduit lumen before stent placement—may predispose to ascending infections. Paralytic ileus remained common (19.8%) despite minimally invasive surgery and ERAS pathways; although all cases were managed conservatively, ileus significantly affects recovery and LOS. Optimizing antiemetic strategies, ambulation protocols, and bowel‐handling techniques may reduce incidence further [17–19]. While minimally invasive approaches have been associated with reduced rates of ileus compared to open surgery in the majority of studies, the incidence remains substantial. Although paralytic ileus represents a non‐life‐threatening complication, its impact on postoperative recovery, patient comfort, and hospital stay should not be underestimated. Genitourinary complications were dominated by ureteral strictures (6.3%), consistent with reported rates of 2%–12% in high‐volume iRARC series [20, 21]. Variability likely reflects differences in anastomotic technique, ureteral handling, stent protocols, and follow‐up duration. Standardizing anastomotic technique, ensuring adequate intraoperative visualization, and optimizing perioperative stent protocols may help reduce the incidence of these complications in the future.

Beyond characterizing morbidity, our findings highlight several opportunities for preventive and mitigation strategies. First, individualized optimization of ERAS pathways may help reduce systemic complications. Elements such as prehabilitation in frail patients, early mobilization tailored to comorbidity burden, structured antiemetic algorithms, and avoidance of excessive intraoperative fluids may reduce rates of postoperative ileus, pneumonia, and thromboembolic events. Cardiovascular complications—one of the most common early high‐grade events in our cohort—underscore the importance of targeted preoperative risk stratification. Incorporating tools such as the G8 frailty score and formal cardiology assessment for patients with unexplored risk factors, along with aggressive optimization of anemia, nutrition, and metabolic comorbidities, may reduce cardiopulmonary morbidity. For infectious complications, particularly UTIs and infected lymphoceles, several technical refinements and prophylactic measures merit consideration. Careful handling of ureteral stents, and earlier stent exchange or removal in selected patients could reduce ascending infections. Likewise, meticulous lymphatic control using the “clip‐and‐cut” technique during PLND may decrease lymphocele formation. Finally, structured postoperative surveillance with early laboratory markers, low thresholds for imaging, and close coordination with infectious disease, cardiology, and interventional radiology services can facilitate timely intervention and prevent escalation to high‐grade complications. These combined strategies illustrate how perioperative pathways can be adapted to the specific morbidity profile of intracorporeal iRARC and may meaningfully reduce the burden of systemic postoperative events.

Importantly, a total of 33 high‐grade complications occurred within 30 days, with 16 additional high‐grade events between days 30 and 90. This distinction highlights that significant morbidity extends beyond the traditional 30‐day window, particularly in patients with urinary tract reconstructions. Thus, reporting complications through a 90‐day period, as recommended by the EAU, provides a more accurate and complete assessment of surgical outcomes.

Our 90‐day rehospitalization rate of 22.3% is slightly lower than contemporary multicenter and population‐based estimates after RC, which typically range from 24% to 40% at 30–90 days (e.g., 28.8% within 90 days in a large series; 39.1% in a national cohort) [22]. The 90‐day mortality of 1.8% in our cohort compares favorably with historical population data (5%–11% overall; increasing with age/comorbidity) and aligns with modern randomized and cohort evidence supporting the safety of iRARC with intracorporeal diversion [23].

While our findings support the feasibility and relative safety of iRARC with intracorporeal diversion, several limitations merit mention. This is a single‐institution, retrospective analysis with a moderate sample size, which inherently limits generalizability. The absence of a direct control group (e.g., open RC or RARC with extracorporeal diversion) restricts the ability to draw comparative conclusions. In addition, selection bias is likely, particularly during the early learning curve phase, when patient selection was more restrictive. Moreover, while perioperative outcomes were robustly assessed, long‐term oncological and functional data were beyond the scope of this study.

## 5. Conclusion

This single‐center analysis confirms that iRARC is feasible and can be implemented with an acceptable safety profile, even during the initial learning curve. Our detailed complication profile reveals that the most significant burden stems from systemic issues, primarily infections and cardiopulmonary events, rather than direct surgical failures. This finding underscores that the success of a cystectomy program depends not only on surgical proficiency but also on robust, multidisciplinary perioperative management involving medical specialists, anesthesiologists, and dedicated nursing care.

## Conflicts of Interest

The authors declare no conflicts of interest.

## Author Contributions

Anas Elyan and Ashkan Mortezavi contributed to study concept and design. Anas Elyan and Laura Wimmer performed data acquisition. Anas Elyan, Ashkan Mortezavi, and Laura Wimmer analyzed and interpreted the data. Anas Elyan and Ashkan Mortezavi drafted the manuscript. Jan Ebbing, Abolfazl Hosseini, and Helge H. Seifert contributed to critical revision of the manuscript for important intellectual content. Anas Elyan performed statistical analysis. Ashkan Mortezavi supervised the study.

## Funding

Open access publishing was facilitated by Universitat Basel, as part of the Wiley–Universitat Basel agreement via the Consortium of Swiss Academic Libraries.

## Supporting Information

Additional supporting information can be found online in the Supporting Information section.

## Supporting information


**Supporting Information 1** Supporting Table 1: Annual case numbers illustrating the transition from open cystectomy to robot‐assisted laparoscopic radical cystectomy with intracorporeal urinary diversion at our institution, covering the period from 2021 to April 2023.

**Supporting Information 2** Supporting Table 2: Detailed overview of the enhanced recovery program implemented following intracorporeal robot‐assisted radical cystectomy.

**Supporting Information 3** Supporting Table 3: Overview of compliance with the European Association of Urology quality criteria for radical cystectomy research. The table outlines essential methodological standards, including data acquisition, follow‐up protocols, and reporting completeness, with corresponding compliance status for the present study cohort.

**Supporting Information 4** Supporting Table 4: Predictors for high‐grade (Clavien–Dindo ≥ IIIa) complications.

**Supporting Information 5** Supporting Figure 1: Flowchart of patient selection for the study cohort.

## Data Availability

The data that support the findings of this study are available upon request from the corresponding author. The data are not publicly available due to privacy or ethical restrictions.
