# Supplementary figures and images for: Comprehensive 90‐Day Morbidity Assessment Following Robot‐Assisted Radical Cystectomy With Intracorporeal Diversion
Source: Adv Urol. 2025 Dec 29;2025:5585494. doi: 10.1155/aiu/5585494 (PMC12746349; doi:10.1155/aiu/5585494)

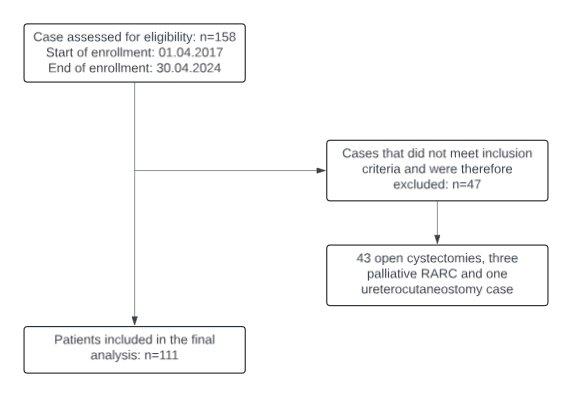

Supplement: Supplementary file 5 — Supporting Information 5 Supporting Figure 1: Flowchart of patient selection for the study cohort. [file AIU-2025-5585494-s001.png]
